# Spared corticospinal neurons activate an endogenous plasticity program after partial CNS injury

**DOI:** 10.64898/2026.09.08.750239

**Published:** 2026-10-22

**Authors:** Matias Murillo, Ciara F O’Brien, Noa Golan, Emma X Yin, Jessica Mariani, Michael P J Deans, Novin Balafkan, Kristen J Brennand, William B J Cafferty

## Abstract

Functional recovery after incomplete spinal cord injury depends substantially on the capacity of anatomically spared neurons to remodel their connections, yet the molecular programs underlying this endogenous plasticity remain poorly understood. To identify and validate these mechanisms, we combined retrograde labeling, unilateral corticospinal tract injury, and spatial transcriptomics in mice, with an *in vitro* human neuron model of neurite regeneration. Injured corticospinal neurons (CSNs) exhibited widespread transcriptional downregulation; the few activated pathways included stress, cell death, and degenerative programs. In contrast, spared CSNs activated a coordinated pro-plasticity program characterized by metabolic, immune, and cytoskeletal remodeling together with suppression of axon growth-inhibitory signaling. Computational network and drug perturbation analyses identified, and human neuronal experiments validated, *ARHGEF12* suppression and the histone deacetylase inhibitor (HDAC) vorinostat as complementary target- and state-based strategies to enhance neurite regeneration. These findings define key components of endogenous plasticity and provide a framework for discovering neural repair targets.

## Full Text Availability

The license terms selected by the author(s) for this preprint version do not permit archiving in PMC. The full text is available from the preprint server.

